# IS SELF-RATED HEALTH A WINDOW ONTO THE BIOLOGY OF HEALTH AND AGING?

**DOI:** 10.1093/geroni/igz038.1264

**Published:** 2019-11-08

**Authors:** Daniel W Belsky

**Affiliations:** Columbia University Mailman School of Public Health, New York, New York, United States

## Abstract

Self-ratings of health predict survival in older adults, suggesting that they capture important information about system integrity. We analyzed epigenetic clock, blood biochemistry, and functional test data alongside participant reports of disability, morbidity, and self-rated health in population-based cross-sectional and longitudinal datasets from the US and UK (total N>50,000) and in a randomized trial of caloric restriction (N=220). We (1) profiled cross-sectional biomarker correlates of self-rated health; (2) quantified residual biomarker associations with self-rated health after accounting for research-participant reports of morbidity and disability and evaluated variation in associations across strata of age, birth-cohort cohort, socioeconomic status, and cognitive functioning; and (3) tested coordinated change in biomarker indices of system integrity and self-ratings of health in response to caloric restriction. Results develop an understanding of self-rated health as a window onto biological processes of aging and highlight important design considerations for future research to illuminate the biological basis of health.

